# Cross-cultural adaptation, reliability and validity of the Spanish version of the upper limb functional index

**DOI:** 10.1186/1477-7525-11-126

**Published:** 2013-07-26

**Authors:** Antonio I Cuesta-Vargas, Philip C Gabel

**Affiliations:** 1 Department of Physiotherapy, Faculty of Health Science, University of Malaga, Malaga, Spain; 2 School of Clinical Science, Faculty of Health, Queensland University of Technology, Kelvin Grove, Australia; 3 Centre for Healthy Activities, Sport and Exercise, Faculty of Science, University of the Sunshine Coast Queensland, Sippy Downs, Australia

**Keywords:** Upper limb, Psychometrics, Outcome measure, Spanish

## Abstract

**Background:**

The Upper Limb Functional Index (ULFI) is an internationally widely used outcome measure with robust, valid psychometric properties. The purpose of study is to develop and validate a ULFI Spanish-version (ULFI-Sp).

**Methods:**

A two stage observational study was conducted. The ULFI was cross-culturally adapted to Spanish through double forward and backward translations, the psychometric properties were then validated. Participants (n = 126) with various upper limb conditions of >12 weeks duration completed the ULFI-Sp, QuickDASH and the *Euroqol Health Questionnaire 5 Dimensions* (EQ-5D-3 L). The full sample determined internal consistency, concurrent criterion validity, construct validity and factor structure; a subgroup (n = 35) determined reliability at seven days.

**Results:**

The ULFI-Sp demonstrated high internal consistency (α = 0.94) and reliability (r = 0.93). Factor structure was one-dimensional and supported construct validity. Criterion validity with the EQ-5D-3 L was fair and inversely correlated (r = −0.59). The QuickDASH data was unavailable for analysis due to excessive missing responses.

**Conclusions:**

The ULFI-Sp is a valid upper limb outcome measure with similar psychometric properties to the English language version.

## Introduction

Health outcome assessment is an important component of patient care. Patient reported outcome (PRO) measures [1,2] are primarily used to objectively reflect a patient’s health or functional status at any given time and to detect changes in this status as a response to an intervention [3]. This assists the clinicians’ understanding of the effects of a condition or disease on a patient’s capabilities, functioning and symptoms [4]. Traditionally, clinical signs and symptoms were used as outcomes and studies that wished to reflect patient health status employed generic quality of life measures. However, over the last two decades region specific PROs that represent the three key body regions, of the upper limb, lower limb and spine have been used more frequently in the assessment of a musculoskeletal patient’s functional status [5]. The Upper Limb Functional Index (ULFI) is a recent example of this. It was initially published in a dichotomous format [6] then updated and modified to a three-point scale [7]. These regional PRO measures are argued to provide greater sensitivity and improved representation of the individual’s functional status than joint or condition specific measures [7-9]. Though various region specific PROs have been used to assess upper-limb functional status, it is accepted that ‘there is no gold standard [8,10-12]. These tools also guide treatment decisions and assess the effectiveness of interventions, including direct comparisons between pre- and post-operative status, and subsequently during rehabilitation [13].

There are several regional upper limb PROs that are advocated and recommended by national associations or organizations around the world for Physical, Occupational and Hand Therapy, Orthopedics and Surgery. This is through their respective institutional websites and subject related Journals. The Disability of Arm, Shoulder and Hand (DASH) [14-16] and the shortened QuickDASH [17] version are two prominent examples. However the DASH has [18] excessive internal consistency, with a documented Cronbach Alpha value >0.95 [6,8,12,19], the recognized upper limit for ‘item redundancy’ or the presence of too many items being too similar to enable a valid change to be detected [20]. The factor structure has also been challenged [21-23] which further questions validity. A questionnaire must provide a single-factor structure so that it can be summated to provide a single or summary score. It cannot be influenced by other constructs such as psychological or emotional status [24,25]. The QuickDASH, as derived from 11extracted DASH items, has also been challenged. The factor structure has not been consistently shown as one-dimensional [7,26-28], which raises concerns on its validity; and it has been found to underestimate symptoms and overestimate disability [29]. Several other regional PROs are also advocated. The Upper Extremity Functional Scale (UEFS) [30] which has been shown to lack reliability and methodological criteria [5,31]. The Upper Extremity Functional Index (UEFI) [32] which is criticized due to it development methodology using a specific workers population in a small data set with a high average age [6,8]. It has been subsequently independently validated [33] but uses a matrix response format which has a high error tendency for completion and scoring [34]. It is also reported to have no advantage over the DASH for measuring clinical change [35]. The Neck and Upper Limb Index (NULI) [36] which has been demonstrated as having item-redundancy from excessive internal consistency [8] and development concerns [37]. There are also a significant number of joint and condition specific scales but these cannot be used regionally as they do not consider the upper limb as a single kinetic chain [8,18].

The ULFI with a three-point option improved both the responsiveness and practicality [7]. It was shown to have strong psychometric properties for reliability, validity, responsiveness, error measurement, and internal consistency that approximated or exceeded those of the DASH and UEFS [6]. The ULFI was shown to have strong psychometric properties for reliability, validity, responsiveness, error measurement, and internal consistency that approximated or exceeded those of the DASH and UEFS [6]. The ULFI’s practical characteristics of brevity, ready transferability to a 100-point scale, ease and rapidity of completion and scoring reinforced the methodological consistency [7,26,38]. This comparative analysis in separate studies has provided scope to suggest the ULFI was preferred to the criterion tools of the DASH [6,17,38], UEFS [6] and QuickDASH [7,26] due to a combination of enhanced psychometric and practical characteristics. A further consideration was that the ULFI has a single factor structure [25] and an acceptable level of internal consistency in all studies.

The ULFI has also been accepted by the international PRO database [39] ‘PROQUOLID’. A Spanish version of the ULFI had not been developed or validated to date. This is significant given that Spanish is one of the five most spoken languages and the second widest geographically [40]. Consequently, a Spanish version of the ULFI (ULFI-Sp) was developed to meet this need. The four published studies to date investigating the ULFI suggest the practical characteristics along with the responsiveness and error range [4,8]^,^ are consistently defined [6,7,26,38]. Therefore the aims of this paper were: to describe the process of translation and cross-cultural adaptation of the original ULFI to Spanish; and to subsequently assess the four critical psychometric properties of reliability, factor structure, internal consistency, and concurrent criterion validity for clinical use with Spanish speakers.

## Materials and methods

### Design

A two stage observational study was conducted involving: initial translation and cross-cultural adaptation of the ULFI [7] to Spanish; then subsequent prospective concurrent completion with a general health questionnaire, the *Euroqol Health Questionnaire 5 Dimensions* (EQ-5D-3 L) [41] and an upper limb regional criterion, the QuickDASH-Sp [42]. A physical therapy outpatients’ population was used for evaluation of the ULFI-Sp’s four critical psychometric properties. TheEQ-5D-3 L was used to clarify the participants’ health status and provide a criterion standard for health comparison. The QuickDASH-Sp was also completed by all participants but there were excessive levels of missing responses that unfortunately rendered the data not useable for analysis and reporting in this study. This may have been partially attributed to the QuickDASH being the final questionnaire in the completion sequence and subsequent patient burden due to the number of questionnaires. All questionnaires were completed and two assessors performed the initial and any subsequent assessments, but were blinded to baseline scores in order to ensure independent collection of outcome data.

### Translation of the ULFI to the “ULFI-Sp”

**Figure 1:**
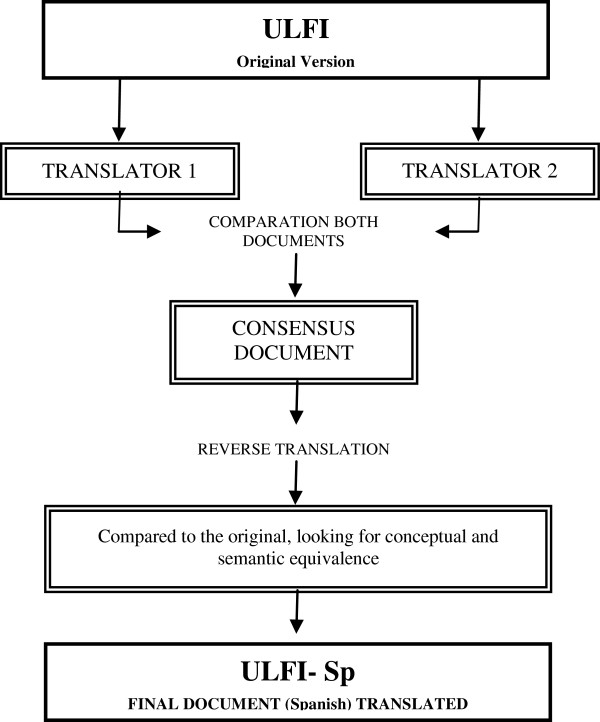
Flowchart of the translation of the Upper Limb Functional Index (ULFI) from English to Spanish.

The primary objective of this aspect of the study was to perform a translation that can ensure the conceptual equivalence of the used terms. Not only a direct and reverse translation methodology was applied, also a specialist in the field as detailed and recommended in the specialized scientific literature (Figure 1) [43,44].

### Participants, setting and procedure

A total of 126 volunteers (49 ± 21 years, 54.8% female) with a variety of upper limb conditions of >12 weeks duration were recruited consecutively from the Physical Therapy Clinic at the Malaga University. Written informed consent was obtained from the patient for the publication of this report. Inclusion criteria were an upper limb injury as diagnosed by the attending medical practitioner. Their presenting conditions and diagnoses were broadly classified into five categories (Table 1). The exclusion criteria were age <18 years and poor Spanish language comprehension as required for the completion of the questionnaires. All participants with eligible criteria completed the three Spanish language versions of the self-administered questionnaires, the ULFI-Sp, the EQ-5D-3 L and the QuickDASH-Sp.

**Table 1:** Demographic characteristics and frequency of diagnosis of the study population

***Characteristic***	***Cases***	***Age (years)***	***p value***
		***Mean (sd)***	
***Study Population***	126	49 ± 21	
**Male**	57 (45.2%)	48 ± 23.2	0.02*
**Female**	69 (54.8%)	51 ± 24.1	
***Dominance***			
**Right hand**	88	49 ± 15.5	0.00*
**Left hand**	48	50 ± 17.8	
***Injured at work***			
**Yes**	51	45 ± 15.5	0.01*
**No**	62	56 ± 19.5	
**Unsure**	13	50 ± 21.5	
***Distribution of upper-limb conditions***	***Area***	***Numbers***	***%***
**Impingement Syndrome**	Shoulder	39	31%
**Shoulder Tendinitis/osis**	Shoulder	32	25%
**Wrist fracture**	Hand	30	24.5%
**Arm fracture**	Arm	13	10%
**Carpal Tunnel Syndrome**	Hand	12	9.5%

* Indicates Significance.

The *ULFI* is a 25-item 3-point response option PRO that is scored by simple addition of the responses then converted to a 100 point percentage scale. Up to two missing responses are permitted [7]. The EQ-5D-3 L is a widely used six item non-disease-specific questionnaire. It has five 3-point response options for different quality-of-life dimensions and a sixth question on overall perceived health-related status. The EQ-5D-3 L-Visual Analogue Scale (VAS) is used to reflect the respondent’s self-rated health status on a 100 mm scale and ranked from ‘Best Imaginable’ (100) to Worst Imaginable’ (0). The EQ-5D-3 L has been demonstrated as valid and reliable in the Spanish population [42]. Reliability was performed using the Intraclass Correlation Coefficients Type 2,1 (ICC_2.1_) test-retest methodology in a randomly selected subgroup of the full sample (n = 35, 46 ± 62 years, 61.1% female). Their presenting conditions were representative of the five categories of the full sample and expressed with a 95% CI using scores on the ULFI-Sp from the participants at baseline and on repetition at day seven.

### Statistics

*Descriptive analyses* were applied to calculate means and standard deviations of demographic variables (Table 1). *Distribution and normality* were determined by one-sample Kolmogorov-Smirnov tests (significance >0.05). *Construct validity and factor structure* were determined through the use of questionnaire principal component analysis (PCA) with the *a-priori* requirements for extraction being the satisfaction of all three points: screeplot inflection point, Eigenvalue > 1.0 and accounting for >10% of variance. We satisfied the recommended minimum ratio of five participants-per-item [24]. *Internal consistency* of the scale items was determined from Cronbach's α coefficients as calculated at an anticipated value range of 0.80-0.95 [20,45]. Student's t-test will be developed to check the items behave the same way for men and women.

An external validation scale of the EQ-5D-3 L and EQ-5D-3 L-VAS was used with bilateral correlations to establish if status had changed and an error range of 0 ± 10% was allowed in determining the test-retest reliability.

The *MDC*_*90*_ analysis was performed as described by Stratford [32]. The standard error of the measurement (SEM) was calculated using the formula: SEM = s√(1–r), where s = the mean and standard deviation (SD) of time 1 and time 2, r = the reliability coefficient for the test and Pearson’s correlation coefficient between test and retest values. Thereafter the MDC_90_ was calculated using the formula: MDC_90_ = SEM × √2 × 1.96.

*Criterion validity* was determined through the concurrent use of the EQ-5D-3 L total score and EQ-5D-3 L-VAS scores with the ULFI-Sp measures. The QuickDASH was unavailable due to excessive completion errors. The Pearson’s r correlation coefficient used the criteria of poor (r < 0.49), fair (r = 0.50-0.74) and strong (r > 0.75) [46].

*Sample size* was determined from the previous ULFI studies [7,8,26] indicating a minimum of 106 patients were required to ensure an 80% chance of achieving the required statiscal power for concurrent validity, internal consistency and factor structure allowing for 15% attrition (*p* < 0.05) [46]. For reliability a minimum of n = 29 was required.

All statistical analyses were conducted using the Statistical Package for Social Science version 17.0 (SPSS 17.0) for Windows and LISREL 8.80 [47].

*Ethical clearance* was approved by the Tribunal of Review of Human Subjects at the University of Malaga.

## Results

### Characteristic descriptive of the participants

The demographic and frequency of diagnosis of the study sample are detailed in Table 1. The ULFI was translated and back translated with consideration of the Spanish cultural linguistic adaptation to provide the new ULFI-Sp questionnaire without language difficulties or other conceptual misunderstanding*.* (Additional file 1). The normative values from ULFI-Sp score were mean and standard deviation of 5.88 ± 5.6 points. The ULFI-Sp showed no missing responses and showed a high degree of internal consistency, as illustrated by the high Cronbach value (α = 0.94) with an individual item range of 0.92 to 0.96. The test-retest reliability was high at (r = 0.93) with an individual range of 0.92 to 0.95. The total score was accounted for, not the individual questionnaire response items. Measurement error was determined from SEM and MDC_90_ being respectively at 3.52% and 8.03%. No significance differences were found between gender in the item responses.

**Figure 2:**
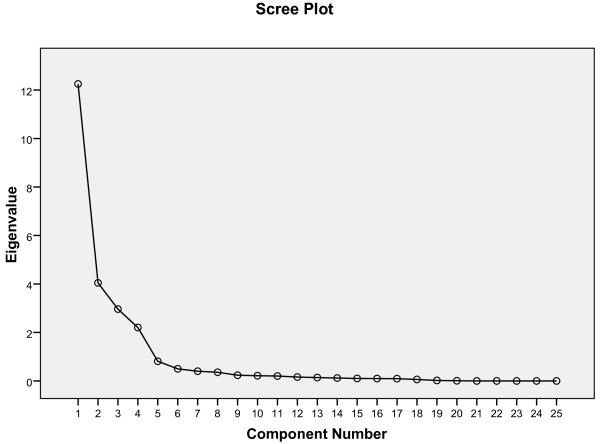
Scree Plot of the exploratory one-factor solution.

The correlation matrix for the ULFI-Sp was determined suitable from the Kaiser-Meyer-Oklin values (0.89) and Barlett’s Test of Sphericity (p < 0.001). This indicated that the correlation matrix was unlikely to be an identity matrix and was therefore suitable for PCA. The screeplot (see Figure 2) indicated a one-factor solution. The factor analysis revealed a satisfactory percentage of total variance explained by the one factor at 48.9%. It was noted that four factors had Eigenvalues >1.0 and accounted for 85.8% of variance; however those with an Eigenvalue >1.0 each accounted for <10% of variance and were shown to be after the screeplot initial inflection point (Figure 2) and consequently not extracted. The item loading for the one-factor solution for the PCA method and average score for each item is shown in Table 2.

**Table 2:** Factor loading items for the one-factor solution, average score and discrimination indices of items

**Question**	**Item**	**Factor loading**	**Item average score**	**Item discr indices**
1	Stay at home most of time	.725	.30	.666^**^
2	Change positions frequently	.842	.44	.824^**^
3	Avoid heavy jobs	.730	.25	.670^**^
4	Rest more often	.914	.17	.886^**^
5	Get others to do things	.914	.12	.886^**^
6	Pain almost all the time	.876	.15	.850^**^
7	Lifting and carrying	.672	.10	.631^**^
8	Appetite affected	.344	.20	.434^**^
9	Walking/normal recreation/sport	.599	.25	.672^**^
10	Home/family duties and chores	.570	.19	.675^**^
11	Sleep less well	.296	.13	.283^**^
12	Assistance with personal care, hygiene	.511	25	.576^**^
13	Regular daily activity work/social	.762	.27	.777^**^
14	More irritable/bad tempered	.344	.18	.466^**^
15	Feel weaker or stiffer	.825	.38	.762^**^
16	Transport independence	.511	.29	.576^**^
17	Arm in shirt sleeve/dressing	.888	.20	.860^**^
18	Writing/using keyboard or mouse	.511	.17	.576^**^
19	Do things at/above shoulder	.295	.10	.209^*^
20	Eating: using utensils	.929	.12	.898^**^
21	Hold or moving dense objects	.891	.18	.868^**^
22	Drop things-minor accidents	.909	.18	.883^**^
23	Use other arm more often	.675	.33	.614^**^
24	Difficult button key coins taps	.825	.52	.762^**^
25	Open, hold, press, or push	.206	.43	.303^**^

** Indicates Significance <0.01.

Criterion validity determined from the relationship between the ULFI-Sp and EQ-5D-3 L (r = −0.59) and EQ-5D-3 L-VAS (r = −0.51) indicated a fair and inverse correlation. The QuickDASH-Sp had greater than >30% of missing responses affecting 41 of the 126 respondent questionnaires. This rendered invalid any reporting of criterion validity or factor structure with this tool.

## Discussion

### Main findings

The ULFI was translated to provide a cross-cultural adaptation to the Spanish language. The translation process ensured the conceptual equivalence of the used terms. This provided accessibility to the ULFI for the second largest geographically used language. The psychometric properties, specifically construct and criterion validity, reliability and internal consistency were determined independently and found to be strong and the single factor structure indicated that a single summated score could be used [25].

The cross-cultural adaptation of the ULFI into Spanish enables clinicians in Spanish speaking settings to compare outcomes following their treatments and interventions affecting the upper limb. The procedure of cross-cultural adaptation of a scale has been used in previous studies for different scales to be applied in the Spanish context [43,44]^.^ The ULFI was translated into Spanish with no difficulty and the process complied with these standardized procedures. It is critical to employ research measures that are valid and reliable but they must also be both culturally and linguistically appropriate.

The one-factor solution that emerged in the factor analysis accounted for a significant proportion of variance and showed evidence that supports the presence of construct validity. A one-factor solution is critical if a PRO is to be used with a single summated score and subsequently reflect the construct for which it is primary used – that of representation of the functional status of the upper limb as a single kinetic chain [25,48]. Any study using confirmatory factor analysis would be advantageous.

Two of the three other critical psychometric properties of the ULFI-Sp were both shown to be high and well supported. Internal consistency analysis showed a level of 0.94 that sits below the accepted 0.95 thresholds for item reducndancy [20]. This notably high level, when taken in context with the presence of the factor structure item loading Eigenvalues above 1.0, would indicate the potential for a shortened version of this tool. This supports the findings of the previous research where redundancy was not present but potential shortening was recommended, perhaps to as low as 10 items [7]. The test-retest reliability or reproducibility was also high with the values (0.92 to 0.95) in-line with those found for the original instrument (0.90 to 0.96) [8].

The criterion validity demonstrated only a fair degree of differential association with the EQ-5D-3 L and EQ-5D-3 L-VAS. This is considered to support the criterion validity of the ULFI-Sp however as it is not as strong as findings in the previous research that used a region specific criterion standard that was advocated as representative of the upper limb kinetic chain. The QuickDASH criterion was not available for criterion comparison due to the excessive number of missing responses. This may have been partially attributed to patient burden as the QuickDASH was the final questionnaire completed. Consequently, this study shows that the ULFI-Sp will be of value in the assessments of patients with upper limb disorders in clinical and research settings.

### Study strengths and weaknesses

The strengths of the study include the prospective nature adequate number of subjects, the inclusion of consecutive patients and the limited selection bias [24,32]. The results for the psychometric properties support the findings of the previous research on the original English version of the ULFI indicating broad cross-cultural adaptions would be appropriate to other diverse cultural and linguistic groups. The ULFI-Sp also provides a means of comparing upper limb health state in Spanish-speaking patients with their English-speaking counterparts in countries with a high Spanish population such as the United States.

The study limitations include the lack of longitudinal data regarding other psychometric properties including responsiveness or sensitivity to change and error scores as a representation of a minimal clinically important difference. The determination of construct validity through the use of factor analysis represents only one possible statistical method of testing. A construct is not restricted to one set of observable indicators or attributes and additional indicators will need to be considered in future research. Similarly, the practical characteristics were not determined. The inability to use the QuickDASH-Sp data collected in the clinical setting due to excessive missing responses, potentially from patient burden due to being the final questionnaire, resulted in no direct comparison with a regional upper limb criterion, a requirement for future studies. Patient burden from completing numerous questionnaires is an area for future consideration. A potential source of bias was that the scales were provided always in the same order. Finally, the inclusion of Hispanic/Latino/ South American participants in future studies could potentially provide confirming or conflicting linguistic information due to the cultural and ethnic difference with respect to the Spanish participants.

## Conclusions

The psychometric properties of the Spanish version of the ULFI are reported here for the first time. The determined values were satisfactory and supportive of the findings of the ULFI as a 3-point scale, particularly in the areas of internal consistency, factor structure and reliability. Consequently, the ULFI-Sp may be useful in Spanish-speaking populations and for making cross-ethnic and cross-cultural comparisons in other English speaking countries with a high Spanish-speaking population.

## Competing interests

The authors declare they have no competing interests.

## Authors’ contributions

All the authors have made contributions to conception of this study. Antonio I. Cuesta-Vargas and Phillip C Gabel participated in the analysis and interpretation of data and were involved in drafting the manuscript or revising it critically for important intellectual content. All the authors have given final approval of the version to be published.

## Supplementary Material

Additional file 1 The Spanish Version of the ULFI. Click here for file
