# Supplementary material for: Cross-cultural adaptation, reliability and validity of the Spanish version of the upper limb functional index
Source: Health Qual Life Outcomes. 2013 Jul 26;11:126. doi: 10.1186/1477-7525-11-126 (PMC3727948; doi:10.1186/1477-7525-11-126)
Supplement: Additional file 1 — The Spanish Version of the ULFI. [file 1477-7525-11-126-S1.doc]

| **UPPER LIMB FUNCTIONAL INDEX (Spanish) FECHA: _________**  **NOMBRE: _______________ LESIÓN: _____________ □ Brazo derecho □ Brazo izquierdo** |
| --- |

**POR FAVOR, COMPLETE:** Sus brazos pueden hacer que sea difícil hacer algunas cosas que hace

normalmente. Esta lista contiene frases que la gente usa para describir esos problemas en ellos mismos.

Piense en usted en los últimos días. **Si una frase lo describe, marque esa casilla. Si no es así, déjela**

**en blanco. Si una frase sólo le describe parcialmente, marque la mitad de la casilla.**

**SOBRE MI/S BRAZO/S:**

1 Me quedo en casa la mayor parte del tiempo.

2 Cambio frecuentemente de postura para aliviar el dolor.

3 Evito hacer trabajos pesados (ejemplo: limpiar, levantar más de 5kg, trabajar el jardín, etc).

4. Paro a descansar más a menudo.

5. Pido a los demás que hagan las cosas por mi.

6. Tengo dolor/problema casi todo el tiempo.

7. Tengo dificultad para levantar y cargar peso (ejemplo: bolsas, compras de hasta 5 kg., etc.).

8. Mi apetito es diferente.

9. El caminar o el hacer mis actividades deportivas y recreativas están afectadas.

10. Tengo dificultad con las tareas normales de la casa y la familia.

11. Duermo peor.

12. Necesito ayuda con mi cuidado personal (ejemplo: la ducha y la higiene).

13. Mis actividades normales diarias (trabajo, actividades sociales) están afectadas.

14. Estoy más irritable y/o de peor humor.

15. Me siento débil y/o rígido.

16. Mi independencia en el transporte está afectada (conducir/transporte público).

17. Tengo dificultad para introducir el brazo en una camisa o para vestirme.

18. Tengo dificultad para escribir o usar el teclado y/o ratón.

19. Soy incapaz de realizar actividades a la misma altura o por encima del hombro.

20. Tengo dificultad para comer y/o usar utensilios (ejemplo: cuchillo, tenedor, cuchara, palillos).

21. Tengo dificultad para coger y mover objetos pesados (ejemplo: tazas, tarros, latas).

22. Tiendo a dejar caer las cosas y/o tengo accidentes menores con mayor frecuencia.

23. Uso el otro brazo más a menudo.

24. Tengo problemas con botones, llaves, monedas, recipientes y tapas de rosca.

25. Tengo problemas para abrir, agarrar, empujar o presionar (ejemplo: palancas, puertas pesadas).

**ULFI PUNTUACIÓN: Para puntuar la parte superior sumar las cajas marcadas:**

**TOTAL (ULFI puntos) 100 Escala: 100 – (TOTALx4) = %**

**MDC (95% CI):** 8.03% o 2 ULFI-puntos. Una puntuación menor a ésta puede ser debido a error.

**Spanish translation courtesy of Dr. Cuesta-Vargas, PhD, Faculty of Health Sciences at the University of Malaga, Spain**
